# Functional tricuspid regurgitation, related right heart remodeling, and available treatment options: good news for patients with heart failure?

**DOI:** 10.1007/s10741-021-10141-6

**Published:** 2021-07-15

**Authors:** Marijana Tadic, Cesare Cuspidi, Daniel Armando Morris, Wolfang Rottbauer

**Affiliations:** 1 Klinik für Innere Medizin II, Universitätsklinikum Ulm, Albert-Einstein Allee 23, 89081 Ulm, Germany; 2 University of Milan-Bicocca, Milan, Italy; 3 Department of Cardiology, Charité – Universitätsmedizin Berlin (Campus Virchow-Klinikum), Berlin, Germany

**Keywords:** Functional tricuspid regurgitation, Heart failure, Tricuspid valve, Outcome, Interventional techniques

## Abstract

Significant functional tricuspid regurgitation (FTR) represents a poor prognostic factor independent of right ventricular (RV) function. It is usually the consequence of left-sided cardiac diseases that induce RV dilatation and dysfunction, but it can also resulted from right atrial (RA) enlargement and consequent tricuspid annular dilatation. FTR is very frequent among patients with heart failure, particularly in those with reduced LVEF and concomitant functional mitral regurgitation. The development of three-dimensional echocardiography enabled detailed assessment of tricuspid valve anatomy, subvavlular apparatus, and RA and RV changes, as well as accurate evaluation of FTR etiology. Due to high in-hospital mortality risk in patients who were operatively treated for isolated FTR, it has been treated only medically for a long time. Percutaneous approach considers mainly transcatheter tricuspid valve repair (edge-to-edge and annuloplasty) and represents a very attractive option for the high-risk patients. Studies that investigated the effects of different devices showed excellent feasibility and safety, followed by significant reduction in FTR grade, improvement in functional capacity and NYHA class, quality of life, and reduction in hospitalization due to heart failure. Some investigations also reported a decreased mortality in FTR patients. Nevertheless, the results of these investigations should be interpreted with cautious due to the small number of participants and relatively short follow-up. The aim of this review was to summarize the existing data about the clinical importance of FTR and FTR-induced right heart remodeling and currently existing therapeutic approaches for treatment of FTR.

## Introduction

**Fig. 1:**
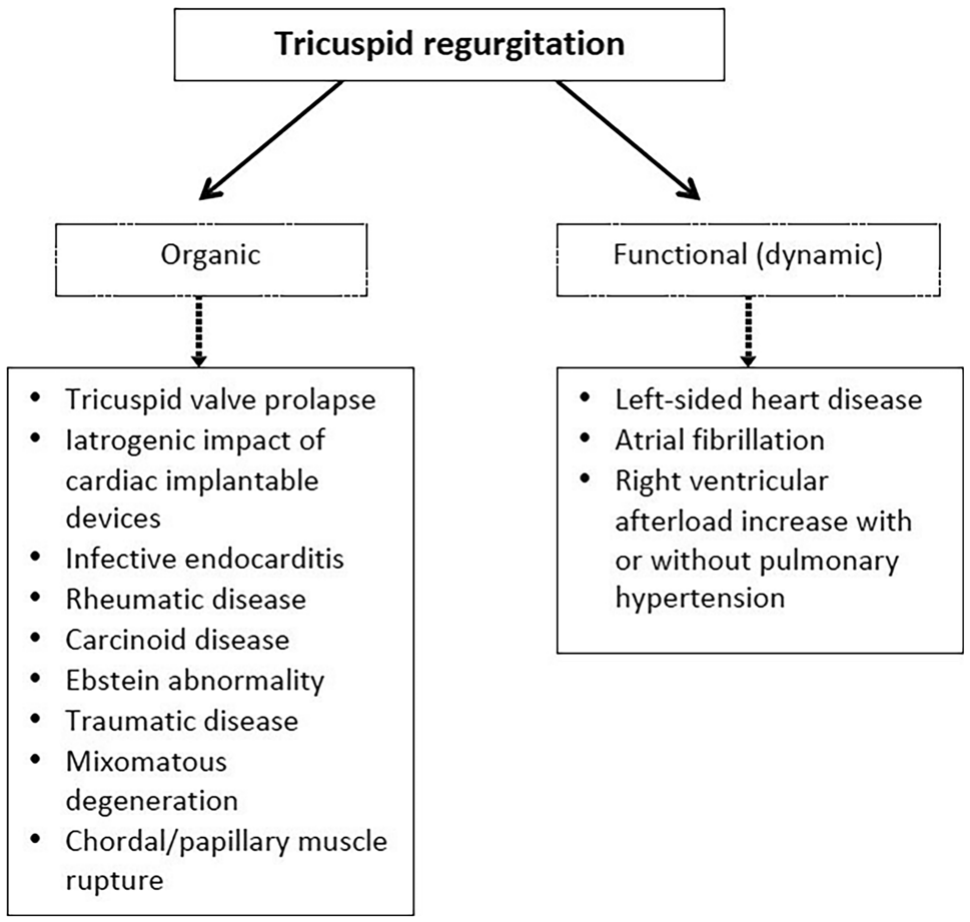
Difference in etiologies between organic and functional tricuspid regurgitation

The importance of tricuspid valve (TV) disorders, at first place regurgitation, has not been considered as important for the outcome and overall survival for a long period of time. In the last decade, our knowledge about TV anatomy, morphology, and function significantly increased due to enormous development of imaging techniques and primarily three-dimensional echocardiography (3DE) [1]. At the same time, growing data of evidence revealed the importance of functional tricuspid regurgitation (FTR) on the outcome in patients with different cardiovascular diseases (CVD) including concomitant valvular disease (aortic stenosis, mitral regurgitation), heart failure, pulmonary hypertension, and even in patients without evident CVD [2–5]. Figure 1 illustrates difference in etiologies that lead to primary (organic) and secondary (functional) tricuspid regurgitation.

FTR is associated with significant remodeling of the right ventricle (RV), which includes the impairment of RV mechanical function, dilatation, and ultimately systolic dysfunction [6, 7]. FTR has been also related with further changes in tricuspid valve anatomy (annular dilatation, higher level of tethering, and tenting) and right atrial (RA) dilatation, which altogether induce the lack of coaptation and deterioration of FTR grade [3, 8–10]. Additionally, RA dilatation represents one of the most important risk factors of atrial fibrillation occurrence, which has an important clinical impact in FTR patients. The triad that includes FTR, RV, and RA remodeling makes “circulus vitiosus,” in which is very difficult to identify what is the cause and what is the consequence, and it is even more challenging to interrupt once when begins.

The current review sought to summarize the existing data about the clinical importance of FTR including FTR-induced right heart remodeling, their impact on the outcome, and currently existing therapeutic approaches for treatment of FTR.

## Tricuspid valve anatomy and FTR

**Fig. 2:**
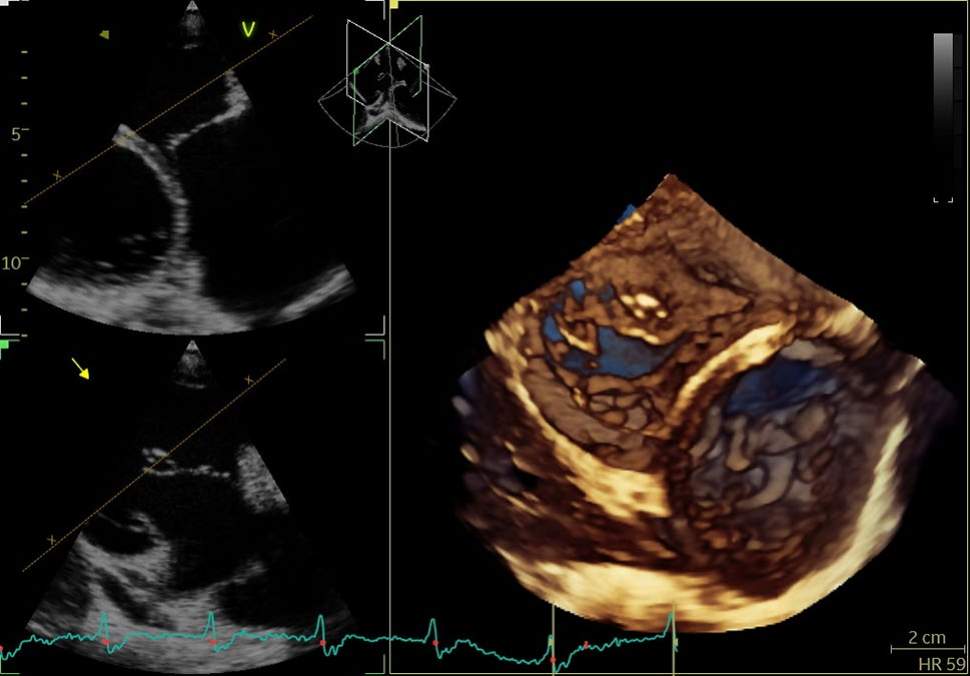
Tricuspid valve in patient with FTR obtained by 3D TTE

Two-dimensional echocardiographic (2DE) TV assessment is challenging due to anatomical position of the tricuspid valve in the chest, specific saddle shape and nonplanar geometry, and 3 leaflets that could significantly vary in their size (surface area and length of the annular attachment). This is why 3DE with possibility to assess TV annulus, leaflets, and subvalvualr apparatus made a revolution in visualization of TV and enabled better understanding of FTR. 3DE datasets provide the overview of the TV from both sides - RV and RA. The later is known as the “surgical view,” and it has been predominantly used for the evaluation of TV changes, whereas ventricular view is used for assessment of tricuspid stenosis and severity of TR (Figure 2). Additional longitudinal planes may be used to estimate the motion of each leaflet, the position, and involvement of chordae tendineae, as well as the papillary muscles, which might be of a great importance during interventional procedures such as edge-to-edge TV repair or interventional TV annulopasty.

Commercially available software dedicated for the assessment of TV anatomy has just been released. However, the majority institutions are still using special cut-planes obtained by slicing the 3DE dataset for all necessary information in the planning of intervention or during procedure. In patients with severe FTR, this approach should be accurate due to flattened TV annulus. However, one should know that normal patients with preserved TV and annulus shape have different values obtained by 3D reconstruction and by direct planimetry of tomographic cut planes [11].

FTR is usually caused by RV dilation and/or dysfunction, which induce leaflet tethering, tricuspid annulus dilation, and ultimately malcoaptation [12]. The most frequent cause of FTR is left-sided valvular or myocardial disease. Prihadi et al recently suggested updated classification of FTR that includes (i) secondary TR induced by significant RV dilation and tethering of the TV leaflets and coaptation gap and (ii) isolated (atrial) TR with dilation of the tricuspid annulus due to RA dilation in the presence of atrial fibrillation–isolated TR [9]. This classification is analogous to the classification that was proposed for functional mitral regurgitation [13].

3DE analysis in healthy subjects showed a nonplanar, saddle-shaped tricuspid annulus [14] with the high points located anteroposteriorly, and the low points located mediolaterally [15]. In patients with FTR, the annulus was more planar, circular, and dilated in the septal to lateral direction [14–16]. Isolated TR is caused by annular dilatation due to RA dilatation, which mainly occurs in patients with atrial fibrillation without associated left-sided heart disease [17, 18]. 3DE assessment demonstrated larger TV annulus area, RA dilatation, and smaller tricuspid leaflet tethering area in patients with chronic atrial fibrillation in comparison to patients with left-sided heart disease [18]. Study that used 3DE evaluation of TV in patients with atrial and ventricular FTR revealed significant differences in TV geometry between these two entities [10]. Anterior-posterior diameter, annular area, and annular orientation were significantly larger in atrial FTR patients, whereas tenting volume and tethering angles were significantly higher in ventricular FTR patients [10]. The results should be interpreted with caution because the authors used software dedicated for 3D mitral valve assessment and not for TV evaluation.

The TR severity may be influenced by the extent of RA remodeling, tricuspid annular dilation, and the type of atrial fibrillation (paroxysmal vs. persistent) [19]. Recent study showed sex differences in pathophysiological mechanisms responsible for FTR progression [20]. Atrial fibrillation was associated with higher risk of FTR in women, whereas higher tenting height was important predictor only in men [21]. Table 1 summarizes grading of TR severity according to the current guidelines [22] and proposed classification which includes terms “massive” and “torrential” TR [23].

**Table 1:** Grading of tricuspid regurgitation severity

American Society of Echocardiography Recommendations [22]					
Severity	Mild	Moderate	Severe		
Quantitative					
EROA (mm^2^)	20	20–39	≥40		
Regurgitant volume (ml/beat)	<30	30–44	≥45		
Proposed new grading system [23]					
	Mild	Moderate	Severe	Massive	Torrential
Vena contracta width (biplane average in mm)	<3	3–6.9	7–13	14–20	≥21
EROA (mm^2^)	<20	20–39	40–59	60–79	≥80
3D vena contracta area (mm^2^)	-	-	75–94	95–114	≥115

*EROA* effective regurgitant orifice area

## The predictive value of FTR

The predictive importance of FTR is the crucial aspect that completely changed our clinical approach to these patients and enabled development of many modern interventional techniques. Study that included 8872 patients with some level of TR reported that 91% of patients had FTR [4]. Almost 45% of all patients had mild, 34% had moderate, and 21% severe TR. Left-sided heart valve disease and LV dysfunction were the most frequent reasons for FTR. The authors only reported that 5-year mortality in patients with severe FTR and pulmonary hypertension was higher than in those without pulmonary hypertension [4]. Unfortunately, the outcome of patients with mild and moderate FTR was not reported. The French study that included only patients with moderate-to-severe and severe TR (85% were patients with FTR) revealed that the 4-year survival was significantly lower than in age- and sex-matched individuals in the general population [21]. Interestingly, patients with FTR without history of LV valvular disease or pulmonary disease had significantly higher mortality risk than those patients with these comorbidities [21]. Another study coming from France included 17,676 consecutive patients who were admitted with a TR diagnosis, mainly FTR due to prior cardiac surgery, ischemic/dilated cardiomyopathy, or mitral regurgitation and showed that in-hospital mortality, 1-year mortality and 1-year mortality or heart failure readmission rates in conservatively treated TR patients were 5.1%, 17.8%, and 41%, respectively [24].

Schneider et al. included almost 30,000 patients with some level of TR, without other valve disease or LV systolic dysfunction, and followed them at least 5 years [25]. Severe TR was present in 790 patients (2.6%), and it was associated with increased 5-year mortality that was more prominent in the patients with pulmonary hypertension [25]. On the other hand, in patients with severe TR without pulmonary hypertension, RV dysfunction was the best predictor of mortality. In those patients with pulmonary hypertension, mortality was independent of the presence or absence of RV dilatation or dysfunction [25].

Some studies demonstrated that FTR severity was associated with worse outcome. During 47-month follow-up, Muraru et al. showed that event-free rate was 14%, 48%, and 93% in patients with severe, moderate, and mild FTR, respectively [2]. Event was defined as death or occurrence of heart failure. The large cohort of patients with degenerative MR (*n* = 5083) reported that trivial FTR existed in 45%, mild in 37%, moderate in 15%, and severe in 3% patients [26]. Survival throughout 10-year follow-up was strongly associated with FTR severity (82% for trivial, 69% for mild, 51% for moderate, and 26% for severe, respectively) [26]. Valvular surgery improved outcome without changing the FTR-associated mortality risk. Data from the Japanese registry of patients with aortic stenosis (AS) included 3815 consecutive patients with severe AS of whom 628 had moderate or severe FTR and 3187 patients with no or mild FTR and reported a poor 5-year outcome (death or hospitalization due to heart failure) in patients with significant concomitant FTR, regardless of the initial treatment strategy (concomitant tricuspid annuloplasty) [5]. Meta-analysis that included 27,614 patients with severe AS undergoing TAVR (6255 with and 21,359 without significant TR) showed that significant TR (moderate and severe) was associated with short-, mid-, and long-term mortality after adjustment for demographic, clinical, and echocardiographic characteristics of the studied population [27].

Significant negative predictive impact of 30-day mortality of moderate and severe FTR was proven also in the patients after non-cardiac surgeries [27]. Interestingly, there was no difference in 30-day risk of heart failure development between patients with significant and non-significant FTR [27]. Five-year survival was significantly worse in patients with significant FTR (moderate and severe) than in patients with mild or without TR, but there was no difference between patients with moderate and severe FTR [28].

The importance of FTR on outcome in patients with heart failure has been well established. Recently published study that included almost half-million US patients with heart failure showed that FTR was related with a significant elevation in mortality risk [29]. Adjusted mortality hazard ratios for patients with mild, moderate, and severe FTR were 1.48 (95%CI 1.44–1.52), 1.92 (95%CI 1.86–1.99), and 2.44 (95%CI 2.32–2.57), respectively [29]. The investigation that included more than 5500 Veterans Affairs patients showed that significant (moderate or severe) TR was associated with increased mortality, regardless of LVEF [30]. For patients with LVEF > 50%, the mortality risk was 1.49 (95%CI 1.34–1.66) and in patients with LVEF < 50%, the mortality risk was 1.54 (95% CI 1.37–1.71), compared to patients with trivial or mild TR [30]. Benfari et al. involved 13,026 patients with heart failure with LVEF < 50% and detected FTR in 88% of participants (33% trivial, 32% mild, 17% moderate, and 6% severe FTR) [31]. Five-year survival significantly decreased with increasing severity of FTR (68 ± 1%, 58 ± 2%, 45 ± 2%, and 34 ± 4%, for trivial, mild, moderate, and severe FTR, respectively) [31].

Meta-analysis of 32,601 patients with a mean follow-up of 3.2 years demonstrated that moderate and severe FTR was associated with a two-fold increased mortality risk compared to no or mild FTR (RR 1.95, 95%CI 1.75–2.17), independently of pulmonary pressure and RV failure [32]. Risk ratio for mortality gradually increased from patients without TR, across those with mild and moderate, to patients with severe TR [32].

## Right ventricular remodeling and its predictive importance in FTR

FTR is associated with RV remodeling—RV dilatation is one of the lead causes of FTR, but once when FTR develops, it only further deteriorates RV structure and function. Viatez et al. included large number of patients TR, of whom even 92.6% had FTR, and reported that RV was dilated in 81.7% of patients with massive/torrential TR, in 55.9% with severe TR, and in 29.3% with moderate TR [33].

**Fig. 3:**
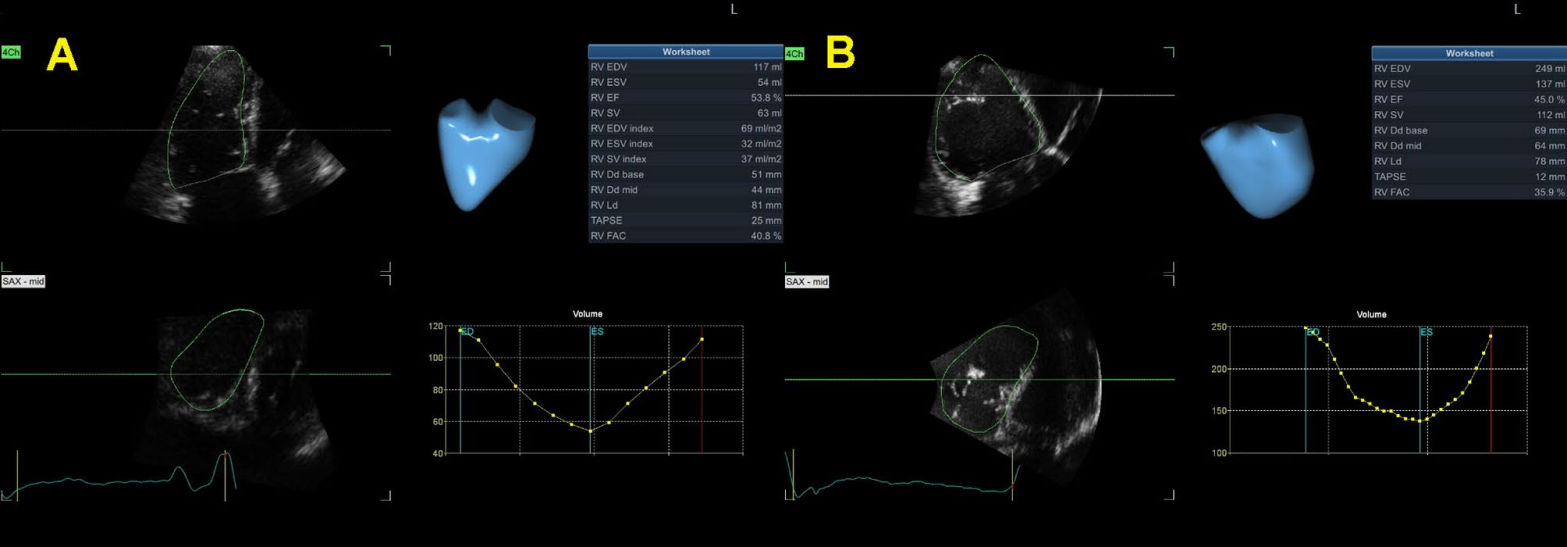
3D evaluation of RV volumes and ejection fraction in two patients with FTR (severe FTR - Panel A, torrential FTR – Panel B)

**Fig. 4:**
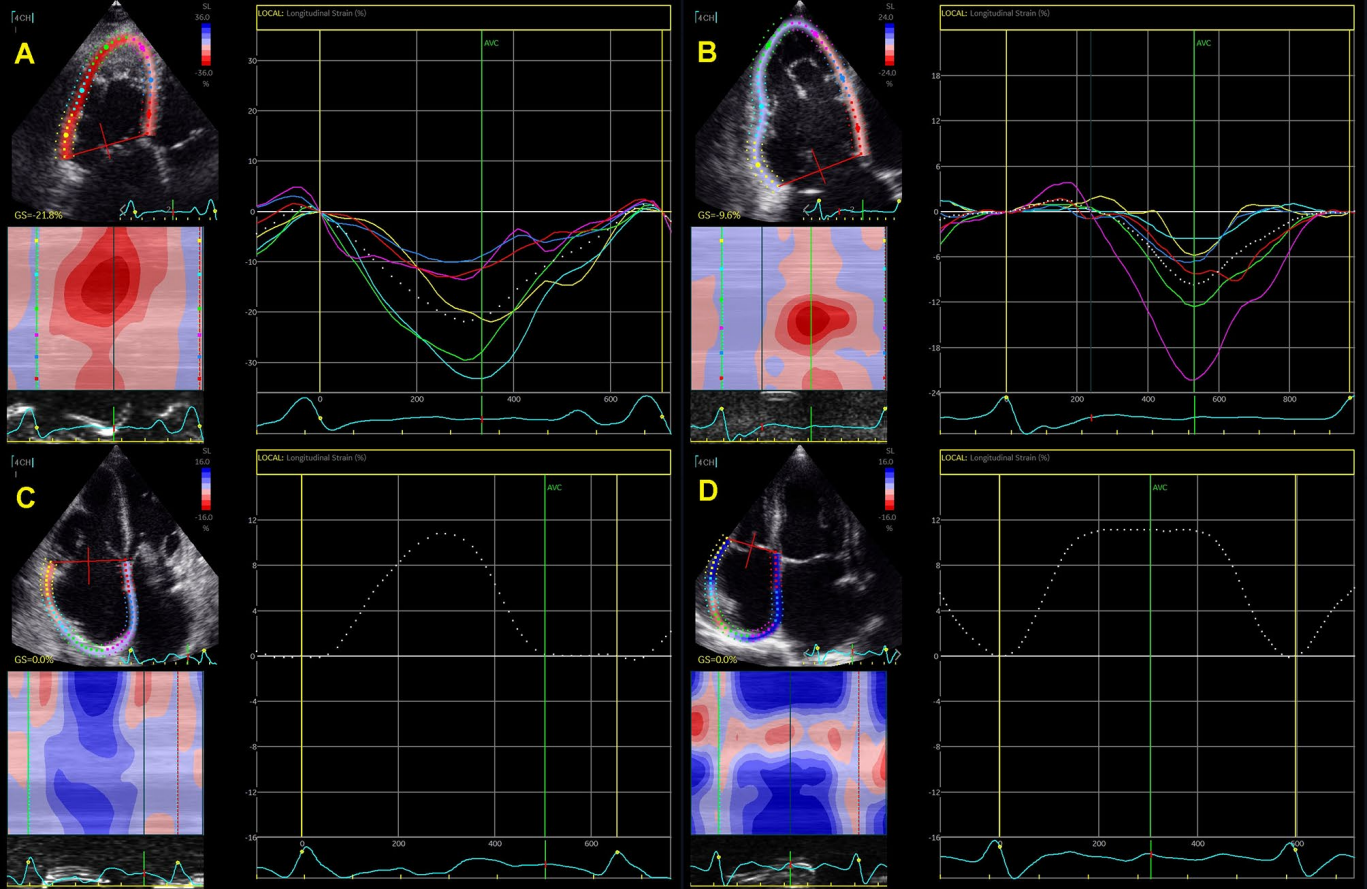
RV and RA longitudinal strain in patients with severe and torrential FTR. Panel **A** Preserved RV longitudinal strain in patient with severe FTR. Panel **B** Severely reduced RV longitudinal strain in patient with torrential FTR. Panels **C** and **D** Reduced RA longitudinal strain in patients with severe and torrential FTR

Recently published study compared RV function and strain between patients with significant and non-significant FTR using 2DE and computed tomography (CT) and reported significantly higher CT-derived RV volumes and lower CT-derived RVEF, 2DE-derived LVEF, and free-wall RV longitudinal strain in patients with significant FTR (moderate and severe) in comparison to those with trace or mild FTR [34]. Utsunomiya et al. reported no difference in free-wall RV longitudinal strain between patients with atrial FTR and controls, even though FTR patients had significantly larger 3DE RV and RA volumes [10]. Nevertheless, patients with ventricular FTR had significantly lower free-wall RV longitudinal strain and larger 3DE RV and RA volumes than patients with atrial FTR [10]. Figures 3 and 4 show significant increase in 3D RV volume and reduction in RV and RA strain in patients with FTR.

Study that included HFrEF patients and followed them for 5 years showed that patients with moderate and severe FTR had significantly larger RV diameter, RV area, and RA volume than those with trace or mild FTR [3]. Tricuspid annular plane excursion (TAPSE) and free-wall RV longitudinal strain were at the same time significantly lower in patients with moderate and severe FTR than in those with trace and mild FTR [3]. Vena contracta, effective regurgitant orifice area (EROA), and regurgitant volume were detected as independent echocardiographic predictors of 5-year mortality [3].

The research that followed for 5.4 years 78 patients who underwent TV surgery for FTR showed that CMR-derived RV end-systolic volume index and the systolic RV mass index were higher in patients with MACCEs than those without them [35]. After adjusting for age and sex, only RV mass index remained a significant predictor of MACCEs and all-cause mortality. After including multiple clinical variables in adjustment, RV mass index remained significantly associated with all-cause mortality [35].

The large study that involved 896 patients with significant FTR who were followed for a mean period of 2.8 years reported significantly higher RV diameters, RV volumes, and RA area in non-survivors than in survivors, whereas parameters of RV systolic function (TAPSE, FAC, and free-wall RV longitudinal strain) were significantly lower in non-survivors than in survivors [7]. Mortality was significantly higher in patients with reduced FAC (<35%), decreased TAPSE (<17 mm), and impaired RV free wall longitudinal strain (>−23%). However, only RV free-wall longitudinal strain was independently associated with all-cause mortality and incremental to FAC and TAPSE [7]. Similar study from Germany reported that mortality in patients with TR increased with increasing of TR level, but they also found that reduced TAPSE (<18.5 mm), decreased FAC (<35%), and impaired free-wall RV longitudinal strain (>-18%) were predictors of mortality in the observed population of 1089 patients with TR [35]. However, reduced free-wall RV longitudinal strain was superior predictor of mortality in comparison with reduced FAC (<35%) and TAPSE in the whole population [36]. Ancona et al. involved only patients with severe TR who were mostly presented with symptoms of RV heart failure and advanced NYHA class [6]. The authors used free-wall and global RV longitudinal strain for reclassification of patients with normal RV systolic function assessed by conventional echocardiographic parameters in patients with impaired RV systolic function and revealed that free-wall RV longitudinal strain >−17% predicted the presence of RV heart failure [6]. However, during follow-up, patients with free-wall RV longitudinal strain <−14% showed a better survival. This indicated that different ranges of free-wall RV longitudinal strain might have different implications in patients with severe TR, and particularly in the prediction of RV heart failure and survival. The study that included 544 FTR patients with a median follow-up of 6 years demonstrated that free-wall RV longitudinal strain ≥−16% was a significant predictor of mortality even after adjustment for clinical and echocardiographic parameters, such as RV size and ejection fraction [37].

Kebed et al. recently proposed introduction of the term “massive” TR in patients with bi-plane vena contracta > 0.92 cm [38] (Table 1). Tricuspid annular and RV size (diameters and 3DE volumes) were larger, and RV free-wall longitudinal strain was significantly lower in the massive group, while there were no significant differences in demographics between patients with severe and massive TR [38]. Patients with massive TR had significantly higher mortality than those with severe TR during 8.3-year follow-up period [38].

## The role of surgery in FTR management

Current European guidelines recommend surgical repair of TV in patients with severe FTR who undergo left-heart valve surgery (class I) or in patients with mild or moderate FTR with dilated tricuspid annulus who undergo left-heart valve surgery (class IIa) [39]. Surgery should be considered in patients with severe FTR who are symptomatic or have progressive RV dilatation or dysfunction, in the absence of severe RV or LV dysfunction and severe pulmonary vascular disease or hypertension, but only in patients who already underwent left-sided surgery and in the absence of recurrent left-sided valve dysfunction (class IIa) [39]. The most important predictors of early FTR recurrence were advanced TV tethering and preoperative FTR severity. For the late FTR recurrence, the most important predictors were advanced TV tethering, repair technique (suture vs. ring annuloplasty, flexible vs. rigid, or semirigid ring), and kidney dysfunction [40].

The investigation that included patients who underwent left-sided valve surgery with or without concomitant tricuspid annuloplasty due to FTR showed a significant reduction in RV diameters and improvement in RV systolic function (FAC) in patients with concomitant tricuspid annuloplasty, whereas RV diameters increased and FAC remained the same in the patients without tricuspid annuloplasty 3 years after operation [41]. The large study that included 5661 patients with TR (49% FMR and 51% primary TR) reported that survival and survival free of heart failure readmission were 75% and 62% at 5 years [42]. Patients with FTR had worse in-hospital mortality than those with primary TR (14% vs. 6%, *p* = 0.004), but presentation was also more severe. Independent predictors of adverse outcome were NYHA Class III/IV, moderate/severe right ventricular dysfunction, and right-sided heart failure signs, whereas TR mechanism was not [41]. Alragni et al. included only patients with FTR (*n* = 713) and showed that there was no difference between types of TV annuloplasty prostheses (rigid rings *vs.* flexible bands) in the management of FTR or change in the degree of TR over time [43].

Pawha et al. included 926 FTR patients who underwent TV replacement or annuloplasty over period of 25 years and found that congestive heart failure, renal failure, previous MV surgery, and TV replacement were independent risk factors for late mortality [44]. Small study that included 43 patients who underwent surgery for TR reported that RVEF and free-wall RV longitudinal strain were not independent predictors of mortality, whereas RV end-diastolic volume was an independent predictor of 5-year mortality [45].

Meta-analysis that investigated the outcomes after surgery for FTR showed that pooled early mortality was 3.9%, and late mortality rate was 2.7%/year, of which cardiac-related mortality was 1.2%/year [44]. Nevertheless, TR remains prevalent even after surgery with the risk of early moderate-to-severe TR at discharge of 9.4%. Mortality and overall TR rate were similar between suture vs. ring annuloplasty, but overall TR rate was higher after flexible ring vs. rigid ring annuloplasty [46]. Other meta-analysis on the same topic showed that ring tricuspid annuloplasty provided better outcomes compared to either suture annuloplasty or not performing annuloplasty [47]. In particular, rigid or semirigid rings provide more stable FTR during follow-up.

## The role of interventional techniques in FTR treatment

Transcatheter TV interventions are fast-developing field in interventional cardiology, and they should serve as an alternative approach for symptomatic patients with high risk for conventional surgery. Currently available devices can be classified according to the therapeutic target in several groups: leaflet devices for edge-to-edge repair, annuloplasty devices, heterotopic caval valve implantation, and transcatheter TV replacement. Most commonly used solutions nowadays are devices for edge-to-edge repair (TriClip from Abbott and PASCAL from Edwards Lifesciences) and Cardioband (Edwards Lifesciences) for tricuspid annuloplasty.

According to the current guidelines, TV surgery is recommended in patients with at least moderate TR who are undergoing left-sided valve surgery (level of evidence I) and in symptomatic patients with severe isolated TR—primary and secondary (level of evidence IIa and IIb) [48]. The optimal timing of TV surgery for asymptomatic or minimally symptomatic patients with severe primary TR is still matter of debate. Transcatheter solutions for TR have not been included in the guidelines yet.

### Coaptation devices

The first device that has been long used for the edge-to-edge treatment of FTR was MitraClip. Study that included 249 TR patients (FTR was present in approximately 90%) reported clinical improvement measured by NYHA class reduction, but not reduction in mortality or rate in hospitalization due to heart failure after 1-year follow-up [49]. Tricuspid regurgitation grade ≥3 was found in 96.8% of patients at baseline and 29.4% at final follow-up. Final NYHA functional class did not differ among TAPSE and sPAP quartiles, even when both low TAPSE and high sPAP were present. The other multicentric study that used MitraClip and PASCAL devices for MV in treatment of patients with severe TR (90% FTR) and found clinical improvement measured by NYHA class, 6-min walking test, and hospitalization rate due to heart failure [50]. Procedural success was related with improved 1-year survival (79% vs. 60%; *p* = 0.04) [50]. Similar results were reported by Cai et al. who compared patients treated with MitraClip at the TV side and those who received only medical treatment [51]. In addition, patients treated with MitraClip had lower mortality during 14-month follow-up [51]. Further analyses showed that patients with pulmonary hypertension and dialysis treatment had worse 1-year outcome after interventional procedure for FTR [52].

The TRILUMINATE was the first study that investigated the device dedicated for treatment of TR (TriClip, Abbott) and showed the safety and effectiveness in reducing TR by at least 1 grade with small 6-month mortality rate (5%) [53]. These results are confirmed after 12-month follow-up with mortality rate of 7.1% and significantly improved 6-min walking test, NYHA class and Kansas City Cardiomyopathy Questionnaire score [54].

**Fig. 5:**
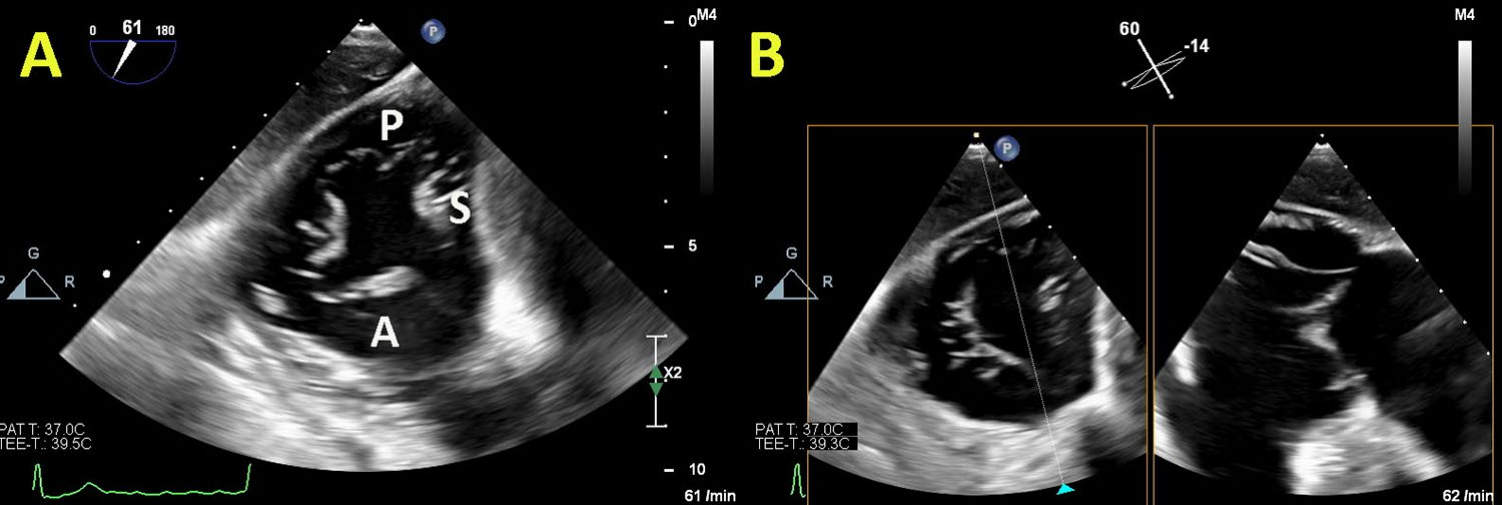
Anatomical assessment of three cusps of the tricuspid valve from transgastric view. Panel **A** “En-face” view with demarcation of anterior (A), posterior (P), and septal (S) leaflet; Panel **B** x-plane view (orthogonal to the first view)

**Fig. 6:**
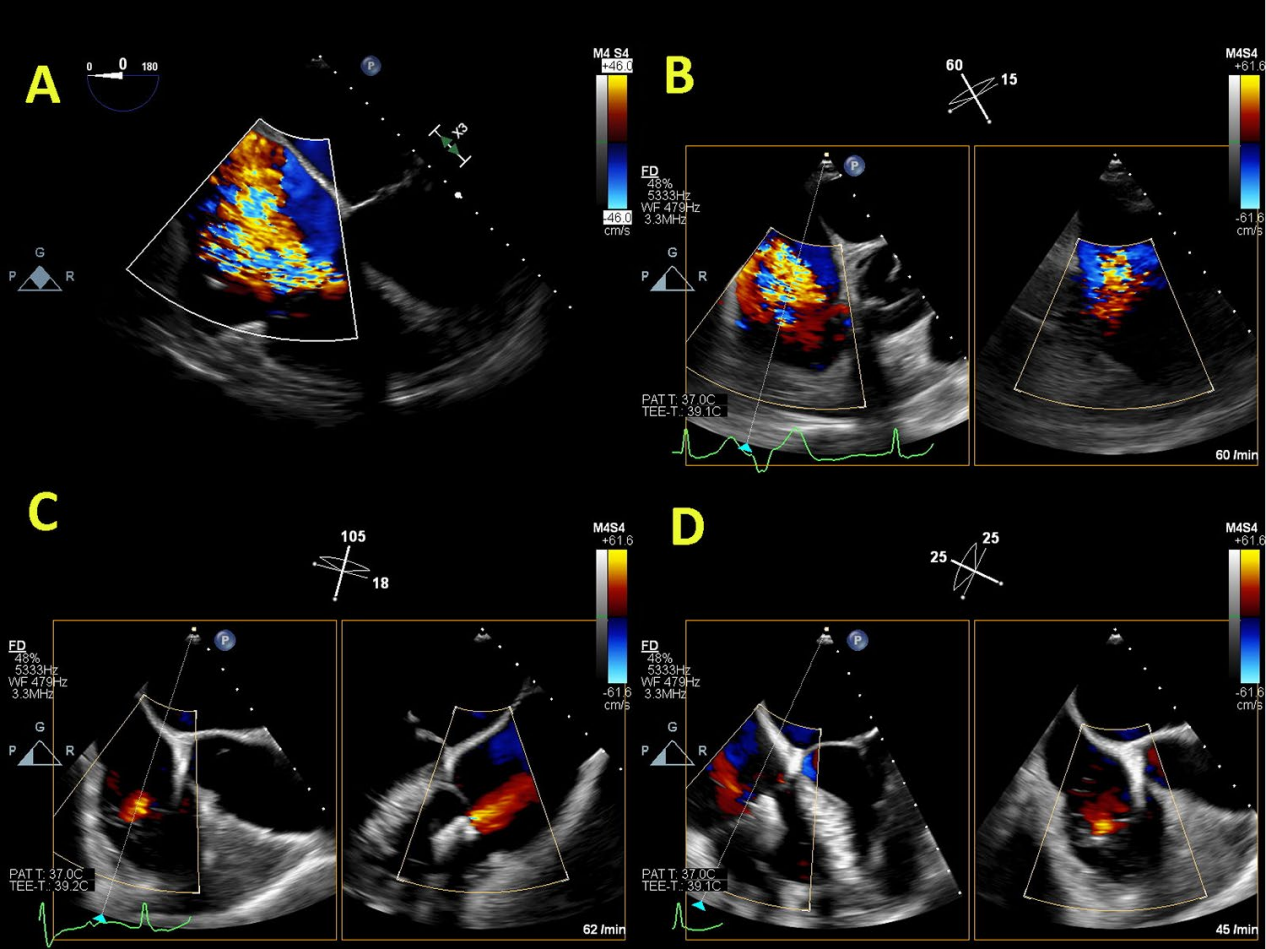
Patients with severe FTR before (Panels **A** and **B**) and after edge-to-edge transcatheter intervention (Panels **C** and **D**)

**Fig. 7:**
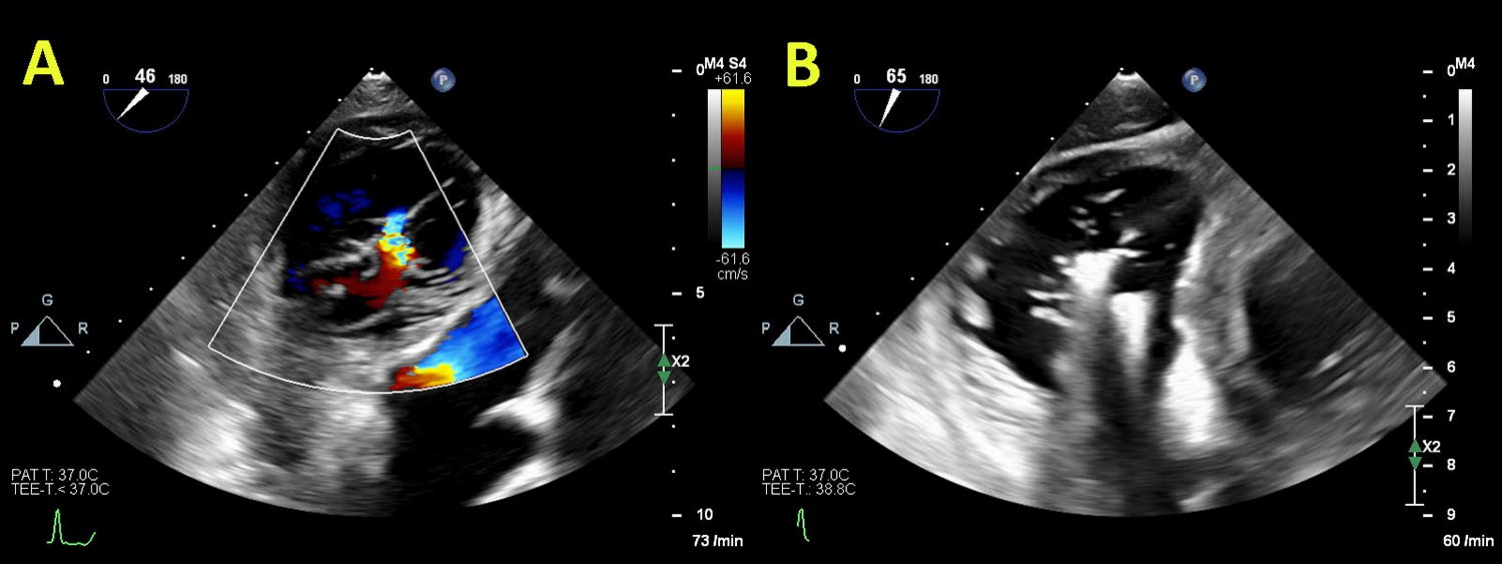
“En-face” view of severe FTR before (Panel **A**) and after (Panel **B**) edge-to-edge transcatheter intervention

The PASCAL system for MV has also been proven as safe and effective for the reduction of TR [54]. The first study showed that mortality was 7.1%, 88% of patients were in NYHA class I/II, with TR grade ≤2+ in 85% and significant improvement in 6-min walking test at 30-day follow-up [55]. Twelve-month outcomes from the use of the PASCAL System in treatment of TR demonstrated high procedural success, acceptable safety, and significant clinical improvement with survival rate of 93%, NYHA class I/II in 90% patients, and significant improvement in 6-min walking test [56]. The early experience about the PASCAL transcatheter valve repair system dedicated for the treatment of TR 30-day follow-up detected 89% patients with NYHA functional class I/II, significant improvement in the mean 6-minute walk distance, and the mean Kansas City Cardiomyopathy Questionnaire score [57]. Figures 5, 6, and 7 illustrate the anatomical assessment of TV in patients with severe FTR before and after edge-to-edge procedure.

The FORMA repair system (Edwards Lifesciences) sought to improve the TV coaptation by reducing the regurgitant orifice with a spacer, which provided a surface for native leaflets coaptation to reduce the orifice area. The device contains a spacer that was advanced through a rail anchored at the septal portion of the RV apex. The first investigations showed good feasibility with a favorable 1-year safety profile [58]. Echocardiographic reduction in TR grade was confirmed in only 48% of patients after 1-year follow-up, but there were significant clinical improvements and reductions in right ventricular dimensions [58]. After 3-year follow-up patients with successful implantation of the FORMA system had sustained improvement in NYHA class, 6-min walk test and mean Kansas City Cardiomyopathy Questionnaire score [59].

### Interventional tricuspid annuloplasty

**Fig. 8:**
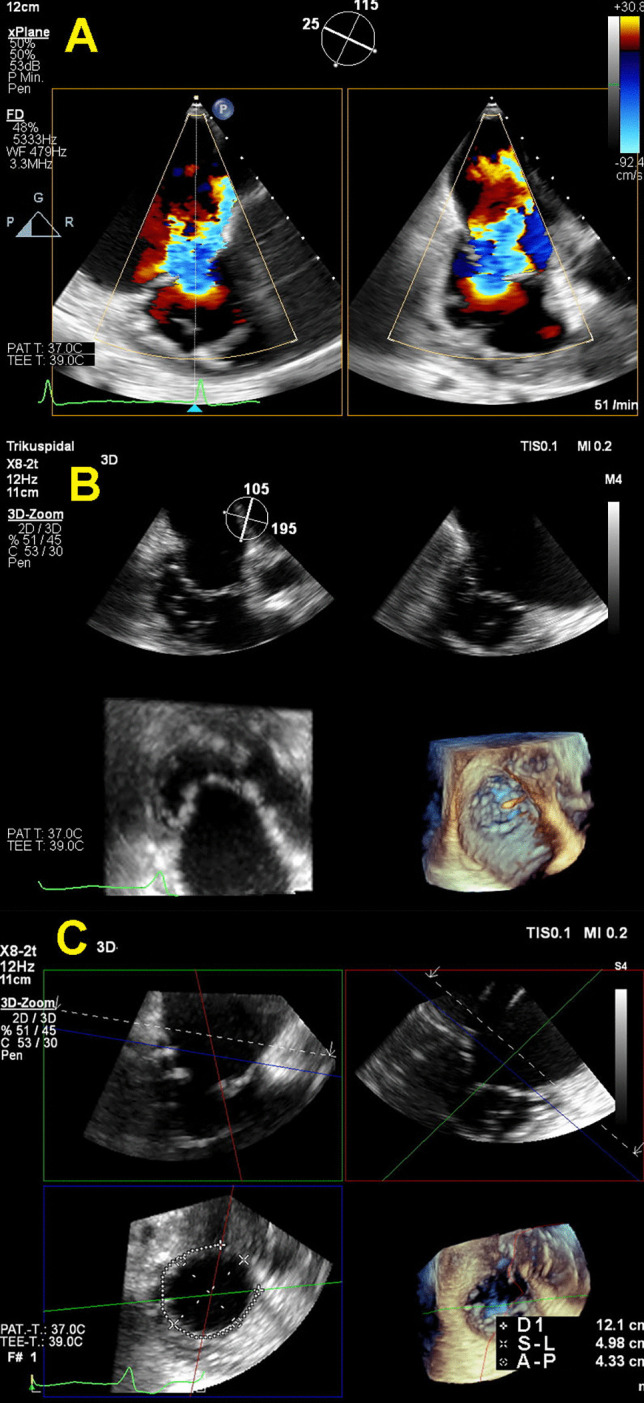
Patient with severe FTR before interventional annuloplasty (Panel **A**), 3D echocardiographic evaluation of tricuspid valve and annulus (Panels **B** and **C**). Footnotes: D1—perimeter of tricuspid annulus between aorta and coronary sinus, A-P—antero-posterior diameter of tricuspid annulus, S-L—septo-lateral diameter of tricuspid annulus

There are several types of tricuspid annuloplasty: suture-based (TriAlign and TriCinch devices), minimally invasive tricuspid annuloplasty with pledget-assisted suture, and ring-based annuloplasty. The last one (Cardioband, Edwards Lifesciences) is the most widely studied and used in the clinical practice so far. Davidson et al. recently reported Cardioband device success of 93% and no death outcome in the first 30 days, with significant reduction in septolateral tricuspid annular diameter, decrease in TR grade, improvement in NYHA functional class, and overall Kansas City Cardiomyopathy Questionnaire score [60]. European experience with this Cardioband is much longer, and the TRI-REPAIR study showed that these improvements in echocardiographic and clinical parameters were sustainable even after 1.7-year follow-up [61]. Figure 8 demonstrates patients with severe FTR who underwent Cardioband and 3D echocardiographic TEE measurements used for planning this procedure.

Computed tomography (CT) has a very important role in the pre-procedural assessment of TV anatomy (annular dimension), as well as the relationship with other important structures, such as right coronary artery (RCA), aorta, and coronary sinus. One of the essential measurements during the procedure is the systematic measurement of the distance between TV hinge point and the RCA. This is determined by CT before procedure and used for the orientation during procedure, in combination with fluoroscopy. Furthermore, specific CT protocols can verify the presence of enough tissue on the tricuspid annulus for the anchors implantation in order to identify the best location for their implantation, which should prevent damage of RCA and other surrounding structures. Another orientation landmarks are aorta and coronary sinus that are easily detected by CT and correspond well with 3D TEE multiplanar reconstruction. New techniques enable fusion of CT, fluoroscopy, and echocardiography during interventional procedure, which provides a better alignment of the delivery system to place the anchors in the predicted position on the tricuspid annulus. However, encouraging results from studies that used interventional techniques to resolve the problem of severe TR showed that this approach should be strongly considered

### Transcatheter TV replacement

Transcatheter TV implantation is still in experimental phase and although there are several devices, there are still not commercially available. The NaviGate valve (NaviGate Cardiac Structures) was one of the first implanted in a patient with a failed tricuspid annuloplasty ring and dilated tricuspial annulus [62]. The EVOQUE system is newly investigated transcatheter TV in 25 FTR patients with an excellent technical success of 92%, no death at 30-day follow-up, 76% of patients with NYHA functional class I/II, and TR grade ≤2 in 96% patients [63]. The GATE system is another recently presented transcatheter TV that was implanted in 30 FTR patients and showed the continued improvement in TR grade between discharge and 30 days [64]. In-hospital mortality was 10%, while at mean follow-up of 127 ± 82 days, 4 patients (13%) had died. Of survivors, 62% were in NYHA class I or II, with no late device-related adverse events [64].

The multicentre TriValve cohort included all existing interventional procedures for treatment of TR (MitraClip, Caval valve implantation, FORMA, Trialign, Cardioband, TriCinch) showed that transcatheter treatment was associated with significant reduction of mortality compared to conservative therapy and might show its best treatment effects in patients with mid-range RV dysfunction TAPSE between 13 and 17 mm) [65]. Meta-analysis that included 454 TR patients treated with different devices (Cardioband, FORMA, MitraClip, PASCAL, and Trialign) showed significant improvement in TR severity, 6-min walk distance, and significant reductions in TV annular diameter, while LV and RV functions did not change significantly [66].

## Conclusion

The importance and interest for FTR has extremely increase over last decade due to growing body of evidence that FTR has significant negative impact on survival in patients with different CVDs and new therapeutic approaches that primarily include interventional methods. According to the current guidelines, severe FTR should be surgically treated only at the time of surgery for left-sided valve disease. Isolated TV surgery is associated with high surgical risk, and therefore, less invasive approach would be very much appreciated. The results from the interventional trials demonstrated a significant improvement in survival, hospitalization rate, and quality of life in FTR patients treated with some of these methods. Several transcatheter techniques are currently available for clinical use and even more are under investigation. Nevertheless, existing studies included small number of patients with limited follow-up and many questions remained to be clarified in the future trials.
